# Low-risk persistent gestational trophoblastic disease treated with low-dose methotrexate: efficacy, acute and long-term effects

**DOI:** 10.1038/sj.bjc.6601422

**Published:** 2003-12-09

**Authors:** F Khan, J Everard, S Ahmed, R E Coleman, M Aitken, B W Hancock

**Affiliations:** 1 Trophoblastic Disease Centre, Academic Unit of Clinical Oncology, The University of Sheffield, Weston Park Hospital, Whitham Road, Sheffield S10 2SJ, UK

**Keywords:** gestational trophoblastic disease, low-dose methotrexate

## Abstract

The aim of this study was to evaluate the efficacy and toxicity of low-dose methotrexate with folinic acid rescue in a large series of consecutively treated patients with low-risk persistent gestational trophoblastic disease. Between January 1987 and December 2000, 250 patients were treated with intramuscular methotrexate (50 mg on alternate days 1, 3, 5, 7) with folinic acid (7.5 mg orally on alternate days 2, 4, 6, 8) rescue. The overall complete response rate without recurrence was 72% for first-line treatment and 95% for those who required second-line chemotherapy. Eight women (3.2%) had recurrence following remission and two (0.8%) had new moles. Two women (0.8%) died of their disease giving an overall cure of 99%. Only 10 women (4%) experienced grade III/IV toxicity during the first course of treatment and 13 women (5.2%) subsequently. Toxicity included mucositis and stomatitis, pleuritic chest pain, thrombocytopenia, uterine bleeding, abdominal pain, liver function changes, rash and pericardial effusion. A total of 59 women (23.6%) required second-line chemotherapy; 48 women had methotrexate resistance, eight had methotrexate toxicity and an empirical decision to change therapy was made in three. In all, 11 women (4.4%) had a hysterectomy before, during or after treatment; 141 women (56.4%) became pregnant following treatment: in 128 (90.7%), the outcome was successful. Methotrexate with folinic acid rescue is an effective treatment for low-risk persistent trophoblastic disease. It has minimal severe toxicity, excellent cure rates and does not appear to affect fertility.

The gestational trophoblastic diseases are complications of pregnancy and comprise a spectrum of conditions that includes complete and partial hydatidiform mole, choriocarcinoma and placental site trophoblastic tumour (PSTT). All have the potential to persist and to metastasise to local or distant structures.

The worldwide incidence of trophoblastic diseases ranges between 0.5 and 8.3 cases per 1000 live births (WHO, 1983), with the incidence in the UK of around 1.5 per 1000 births (Bagshawe *et al*, 1986, Tham *et al*, 2003). In Sheffield, there are around 400 patients registered annually with a molar pregnancy. Most patients have no further problems following evacuation of the mole, but about 5% require chemotherapy for persistent disease (Sheridan *et al*, 1993).

Low-dose methotrexate with folinic acid rescue, as described by Bagshawe *et al*, (1989), has been the standard treatment for ‘low-risk’ disease at the Sheffield Trophoblastic Tumour Centre for many years. In 1987, we modified our approach so that all patients who fell into the Charing Cross scoring system ‘low’- and ‘medium’-risk groups (Newlands 1997) were given low-dose methotrexate as first-line therapy. This study reports on the efficacy and toxicity of treatment in this group of patients.

## PATIENTS AND METHODS

Sheffield is one of three supraregional screening centres and one of two treatment centres in the UK for the registration, specialist management and follow-up of gestational trophoblastic diseases.

All patients diagnosed with a molar pregnancy in Northern England and North Wales (population approximately 22 million) are registered at the Sheffield centre, but initially managed by their local gynaecologist with uterine evacuation; hCG is monitored regularly at Sheffield. Between January 1987 and December 2000, 5613 patients were registered; 250 patients were treated for low-risk persistent trophoblastic disease. During the same period, 82 high-risk patients were treated with intensive multiagent therapy (methotrexate alternating with etoposide and dactinomycin) (Dobson *et al*, 2000).

The criteria for treatment of trophoblastic disease were:

1. hCG levels >20 000 IU l^−1^ after uterine evacuations;
2. Static or rising hCG levels after uterine evacuations;
3. Persistent uterine haemorrhage with elevated hCG level;
4. Persistent hCG elevation, 6 months after uterine evacuation;
5. Pulmonary metastases with static or rising hCG levels;
6. Metastases in liver, brain or gastrointestinal tract;
7. Histological diagnosis of true choriocarcinoma.

Patients were assessed at Weston Park Hospital with a full history and physical examination, endocrine and bio-chemical blood tests, full blood count, serum *β*hCG levels, histopathological review of the products of evacuation, chest radiograph, ultrasound of the pelvis and computerised tomography of the thorax and upper abdomen. This information was used to assign a risk score to each patient using the Sheffield modification of the Charing Cross system (Table 1

**Table 1:** Charing cross hospital prognostic scoring system

**Variable**	**0**	**1**	**2**	**6**
Age (years)	⩽39	>39		
Antecedent pregnancy (AP)	Mole	Abortion/unknown	Term	
Interval between AP to treatment (months) hCG (IU l^−1^)	<4	4–6	7–12	>12
	10^3^–10^4^	<10^3^	10^4^–10^5^	>10^5^
ABO blood group (female × male)		A × O	B × O or O	
		O × A	AB × A or O	
		O or A × unknown		
Number of metastases		1–4	4–8	>8
Site of metastases	Lungs, vagina	Spleen, kidneys	Gastrointestinal tract, liver	Brain
Largest tumour mass	<3 cm	3–5 cm	>5 cm	
Previous chemotherapy			Single drug	Two or more drugs

hCG=human chorionic gonadotrophin; Charing Cross System – low risk: 0−5, intermediate risk: 6−9, high risk: >9. Sheffield modification – low risk: 0−7, high risk: >7.

). Patients were continued on chemotherapy for 6–8 weeks after reaching complete remission (CR); that is, a normal serum *β*hCG.

Low-risk patients received intramuscular methotrexate 50 mg on alternate days (1, 3, 5, 7) with 7.5 mg of oral folinic acid rescue on alternate days (2, 4, 6, 8), 24 h after the methotrexate, with a 7-day break between courses. Strict compliance was ensured by mandatory attendance at Weston Park Hospital for all but a very few selected cases. The first course of treatment was administered as in-patient therapy. Serial serum *β*hCG values were measured as a basis for further treatment. Patients who experienced severe toxicity or whose *β*hCG levels had plateaued or were rising were changed to second-line (intravenous) chemotherapy (Dobson *et al*, 2000), consisting of etoposide and dactinomycin in combination, or methotrexate, etoposide and dactinomycin in combination, or single-agent dactinomycin.

Patients cured on first-line treatment alone were followed up for a period of 5 years with blood and urine hCG analyses. Those who went on to have second-line treatment or who had recurrent disease are followed up for life.

The case notes and database records of all women treated with first-line intramuscular low-dose methotrexate for low-risk trophoblastic disease were studied. The following information was collected: number of evacuations, time from evacuation to treatment, pretreatment serum *β*hCG, site and number of metastases, FIGO stage (FIGO, 1992) (Table 2

**Table 2:** FIGO (1992) staging system for gestational trophoblastic disease (GTD)

*Stage*	
*I*	Disease confined to the uterus
*II*	Beyond uterus but limited to genital structures
*III*	To lungs with or without known genital tract involvement
*IV*	Other metastatic sites
*Sub stage*	
*A*	No risk factor
*B*	One risk factor
*C*	Two risk factors
*Risk factors*	
*1.*	HCG>100 000 U l^−1^
*2.*	Duration from termination of antecedent pregnancy to diagnosis more than 6 months

), risk score, number of cycles received, first course and subsequent toxicity according to the ECOG Committee Toxicity Criteria (Oken *et al*, 1982), reasons for change of treatment, outcome and post-treatment fertility.

The clinical features of the patients are summarised in Table 3

**Table 3:** Clinical features of patients treated

Age (years)	
⩽39	226 (90.4%)
>39	24 (9.6)
Antecedent pregnancy	
Mole	234 (93.6)
Term	1 (0.4%)
Termination	2 (0.8%)
Ectopic pregnancy	5 (4 molar ectopics, 1 not known) (2%)
Other (unspecified/no molar tissue)	8 (3.2%)
Evacuations	
Mean	2.16
Median	2
Time from first evacuation to treatment (months)	
<4	169 (67.6%)
4−6	51 (20.4%)
6−12	24 (9.6%)
>12	4 (1.6%)
No evacuation	2 (0.8%)
*β*hCG (IU l^−1^)	
10^3^−10^4^	89 (35.6%)
<10^3^	59 (23.6%)
10^4^−10^5^	95 (38%)
>10^5^	7 (2.8%)
Number of metastases	
Nil	178 (71.2%)
1−4	53 (21.2%)
4−8	16 (6.4%)
>8	2 (0.8%)
Unknown	1 (0.4%)
Methotrexate courses	
Mean	8
Median	7
Range	1−14

. The age range was 15–57 years (median 27 years). The median time from evacuation to treatment was 94 days (range 7–1149 days). The details of 28 women (11%) who were treated for more than 6 months after evacuation are summarised in Table 4

**Table 4:** FIGO stage of patients treated with first-line and salvage chemotherapy

**FIGO stage**	**Number of patients**	**Cured with first-line treatment**	**Cured after salvage chemotherapy**
*I*	178 (71.25)	145	33
*II*	1 (0.4%)		1
*III*	70 (28.0%)	42	26^a^
*IV*	0		
*Unknown*	1 (0.4%)	1	

a Two patients died of chemoresistant disease.

. Four patients from this group required second-line chemotherapy and one died. The range of serum *β*hCG levels at assessment was 16–803 000 IU l^−1^ (mean 23 736). Outcome was evaluated in all patients and toxicity in 244. At assessment, 178 patients (71.2%) had no detectable disease outside the uterus; the other 72 (29%) had lung metastases only. The primary indication for treatment was persistently elevated hCG level in 235 patients, vaginal bleeding in 14 and intra-abdominal bleeding in one.

## RESULTS

### Response to treatment

All patients received at least one full course of methotrexate (range 1–14, median 7). A total of 59 (23.6%) patients had second-line salvage chemotherapy. All women have been followed up for at least 1 year (range 1–16 years, median 5 years). Complete remission with first-line methotrexate was achieved in 181 (72%) patients. In those who had first- and second-line treatment, CR was attained in 238 (95%). In all, 145 (81%) of 178 FIGO stage I patients achieved CR with first-line treatment compared to 42 (60%) of 70 stage III patients (Table 4).

A total of 48 had methotrexate-resistant disease, eight had severe methotrexate toxicity and an empirical decision to change treatment was made in three. Of the eight patients with severe toxicity, two developed abnormal liver function, one had severe vaginal bleeding, three had pleuritic chest pain, one had abdominal pain and one a pleural effusion.

**Figure 1:**
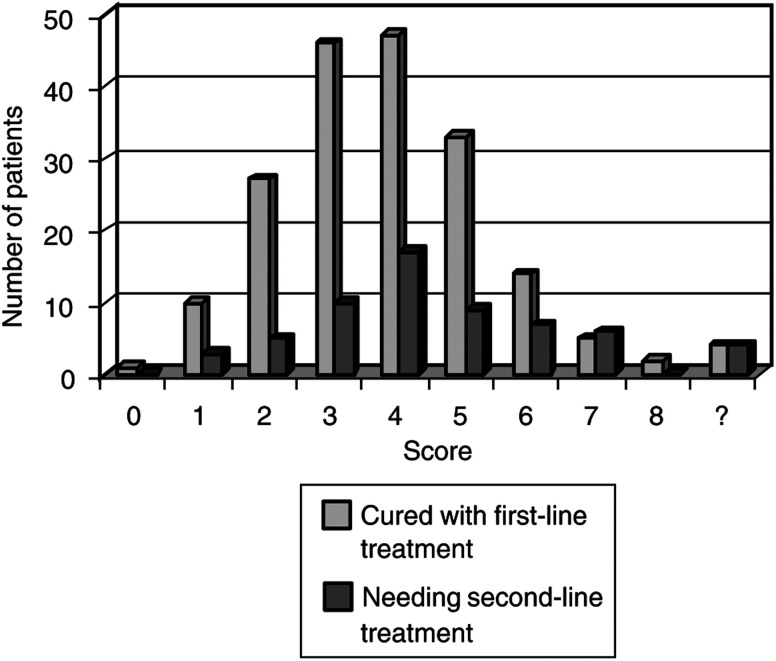
Number of patients cured with first-line treatment compared with those needing second-line treatment, according to risk score.

The proportion of patients requiring second-line chemotherapy increased with risk score, but this was not statistically significant (Figure 1

). Two patients (0.8%) had third-line treatment due to resistant disease and metastases; both patients died of the disease. The presenting risk scores in these two women were 4 and 7.

Following CR, eight women (3.2%) had recurrent disease and two women (0.8%) had new moles. Three women had recurrence within a year, four within 2 years and one within 3 years; six of these patients had recurrence following first-line chemotherapy and two patients had recurrence following first- and second-line chemotherapy. Of the two women who had new moles, one had two subsequent molar pregnancies and is thought to have a genetic predisposition to molar pregnancies. The other woman had no further pregnancies following her second mole. Neither woman required chemotherapy for their subsequent molar pregnancies. One patient died following a road traffic accident, in remission 3 years after finishing methotrexate chemotherapy. The overall survival of all patients was 98%, with 247 patients alive and well.

In all, 17 (6.8%) women experienced delays during treatment; six women defaulted treatment, 10 experienced toxicity (myelosuppression, severe bleeding, biochemical changes) and in one there was no record of why treatment was delayed.

### Toxicity

Toxicity was evaluated for the first and subsequent courses of methotrexate (Table 5

**Table 5:** Side effects from methotrexate treatment^a^

	**First course of treatment**		**Subsequent courses**	
**Side effect**	**Grade I/II**	**Grade III/IV**	**Grade I/II**	**Grade III/IV**
Anaemia			2 (0.8%)	
Neutropenia		1 (0.4%)	1 (0.4%)	2 (0.8%)
Thrombocytopenia		1 (0.4%)	1 (0.4%)	1 (0.4%)
				
Nausea	19 (7.6%)		37 (14.8)	
Vomiting	5 (2%)		12 (4.8%)	
Abdominal pain	21 (8.4%)	2 (0.8%)	34 (1.6%)	1 (0.4%)
Abnormal liver function	1 (0.4%)	1 (0.4%)	3 (1.2%)	
				
Mucositis/stomatitis	61 (24.4%)	1 (0.4%)	65 (26%)	2 (0.8%)
Sore/bleeding gums	2 (1.6%)		8 (3.2%)	
Epistaxis	6 (2.4%)		3 (1.2%)	
Blepharitis/conjunctivitis	61 (24.4%)		60 (24%)	
				
Pleuritic chest pain	22 (8.8%)		60 (24%)	3 (1.2%)
Shortness of breath	1 (0.4%)		5 (2%)	
Pleural effusion				1 (0.4%)
				
Skin rash	8 (3.2%)	1 (0.4%)	2 (0.8%)	1 (0.4%)
				
Vaginal bleeding	49 (19.6%)	4 (1.6%)	38 (15.2%)	2 (0.8%)

a In all, 92 (36.8%) had no toxicity during their first course; 67 (26.8%) had no toxicity during subsequent courses.

). A total of 158 (63.2%) women experienced first course toxicity compared with 183 (73.2%) during subsequent courses. In all, 10 women (4%) during the first course, and 13 women (5.2%) during subsequent courses, experienced grade III/IV toxicity.

#### Haematological

Two women (0.8%) had grade III/IV myelosuppression during the first course. Grade I/II and Grade III/IV myelosuppression occurred during subsequent courses in four (66%) and three (1.2%), respectively. There were no dose reductions due to myelosuppression; however, four women had treatment delays.

#### Other toxicities

Blepharitis, conjunctivitis, stomatitis and mucositis were the most common side effects. Only one woman during the first course and two women during the subsequent courses developed grade III/IV mucositis and stomatitis.

Pleuritic chest pain was also commonly experienced. Three women (1.2%) had their treatment changed during subsequent courses due to grade III/IV chest pain. One patient during the first course and five patients during subsequent courses developed mild shortness of breath. Two of these patients had shortness of breath associated with pleuritic chest pain. One woman developed a pericardial effusion (Forbat *et al*, 1995).

In total, 49 women (19.6%) experienced grade I/II vaginal bleeding during the first course and 38 women (15.2%) during further courses. Four women during the first course and two women during subsequent courses had grade III/IV bleeding. Three (1.2%) women required hysterectomy during treatment.

Mild nausea was a common feature during both first and subsequent courses. One woman had treatment changed due to abdominal pain, thought to be due to peritoneal toxicity (Sharma *et al*, 1999). Grade I/II epistaxis was noted for six women during the first course and in three women during subsequent courses. After course one, one woman had grade I/II liver function changes; one woman had grade III/IV changes and was changed to second-line treatment. Three women had grade I/II liver function changes during further courses. One woman had a grade III/IV rash and had her treatment changed. Alopecia did not occur.

#### Hysterectomy

A total of 11 women (4.4%) had a hysterectomy before, during or after treatment. Four women had uterine rupture or severe bleeding before treatment, three women had severe bleeding during treatment, and following treatment four women had either irregular bleeding or fear of recurrence.

#### Long-term effects

Following treatment, 141 women had one or more further pregnant events; 128 (90.7%) of these were full-term normal pregnancies. There were 21 miscarriages, 11 terminations and one ectopic pregnancy. Only two women (0.8%) had new molar pregnancies. There is no information on long-term fertility status in 98 women (39.2%). There have been no recorded second malignancies.

## DISCUSSION

It is difficult to compare treatment results for persistent gestational trophoblastic disease (GTD) across the world because of the heterogeneity of patient groups selected for single- or multiagent chemotherapy, and because of the wide variety in chemotherapy regimens used. Part of the problem stems from the use of different criteria by various centres for starting treatment and different staging or prognostic scoring classifications. However, in general, patients are selected for therapy on the basis of whether they have metastatic disease and whether they are at low or high risk of persistent or recurrent disease. Patients adjudged as low risk or without metastases have usually been given single-agent regimens (most often methotrexate or dactinomycin). It is hoped that there will be further standardisation with the recent introduction of the revised FIGO/WHO staging/scoring system (FIGO, 2002).

Since the initial experience of using methotrexate in the treatment of trophoblastic disease in the 1950s, it has proved to be an active agent with minimal severe toxicity in the treatment of low-risk patients. Comparisons between various methotrexate regimens and with alternative agents can be made (on efficacy, toxicity and cost effectiveness), both in curing disease and preserving fertility.

The UK has a more conservative approach to initiating treatment for persistent disease than the US; 5–8% of UK and up to 20% of US patients receive treatment. A widely used US protocol recommends that if hCG levels increase or plateau over three consecutive weeks, immediate assessment and treatment for post molar disease are indicated (Berkowitz and Goldstein, 1996. In the UK, treatment is expectant and patients may be followed with serial hCG levels for up to 6 months (Gillespie *et al*, 2000). A recent proposal is that persistent gestational trophoblastic neoplasia should be diagnosed if the plateau of hCG lasts for 4 weeks or more, there is a rise of hCG on three consecutive weekly measurements over a period of 2 weeks or longer, there is a histological diagnosis of choriocarcinoma or the hCG level remains elevated for 6 months or longer (FIGO, 2002). In the UK, each course of chemotherapy is expected to give around a 50% decrease in serum hCG levels. Further treatment is given until complete remission and then for up to 8 weeks following. In contrast, in the US uncomplicated cases, further treatment is often withheld after the first course if the hCG is falling.

Goldstein *et al* (1998), in an evaluation of the revised FIGO (1992) staging, concluded that single-agent chemotherapy is the preferred therapy in patients with nonmetastatic GTD who desire to preserve fertility, and that patients with low-risk metastatic disease involving the pelvis with or without lung metastases (low-risk FIGO stage II, III) also respond well. In total, 351 of 377 (95%) of their FIGO stage I patients were cured by various different single-agent (methotrexate or dactinomycin) regimens. Single-agent chemotherapy also produced remissions in 55 out of 66 (87%) and 13 out of 17 (76%) of low- and medium-risk patients with metastatic disease. Other authors have also reported remission rates of over 80% for this group of patients (Dubeshter *et al*, 1987; Ayhan *et al*, 1992; Dubuc-Lissoir *et al*, 1992).

For many years UK centres have used the low-dose intramuscular methotrexate regimen. Initial reports on 487 patients from Charing Cross (Bagshawe *et al*, 1989) and 115 from Sheffield (Dorreen *et al*, 1988) confirmed its efficacy, although it was noted that up to 30% of patients needed second-line treatment due to resistance or, less frequently, toxicity. In the recent Charing Cross report of use of this regimen for low-/intermediate-risk patients, 33.2% of 485 patients required a change in treatment; however, overall survival was 100% (McNeish *et al*, 2002).

Methotrexate by intravenous infusion has also been evaluated as a treatment option. One protocol (Berkowitz *et al*, 1990) administered 100 mg m^−2^ by intravenous infusion over 30 min followed by 200 mg m^−2^ over 12 h. After 24 h, 15 mg of folinic acid was given intravenously every 12 h for four doses. In nonmetastatic disease, CR was achieved in 68.7% of patients compared with 90.2% in patients on the 8-day intramuscular regimen. None of the patients given methotrexate by infusion developed significant biochemical toxicity. One patient developed a severe rash. On the 8-day regimen, 24.7% of patients developed side effects including myelosuppression, pleurisy, nausea and vomiting and in particular hepatotoxicity. In a more recent study, the methotrexate infusion was given without folinic acid rescue in patients with or without lung metastases (Wong *et al*, 2000). In all, 91.5% of patients achieved CR on first-line therapy alone. Mild nausea affected five patients and one woman had severe myelosuppression, rash, stomatitis and pharyngitis, although interestingly, she had nontoxic postinfusion methotrexate levels. In both these studies, dactinomycin was added to subsequent infusions in patients with resistant disease. However, both studies involved few patients (32 and 59, respectively); additionally, the amount of drug being delivered by infusion was close to twice that given to patients on a low-dose intramuscular methotrexate and folinic acid protocol.

Five-day methotrexate regimens (using both intramuscular or intravenous routes) have also been evaluated (Soper *et al*, 1994; Lurain and Elfstrand, 1995). For example, with 0.4 mg kg^−1^ day^−1^ intramuscularly for 5 days, repeated at 12–14 days, in 52 patients with metastatic disease, 60% achieved primary remission; second-line chemotherapy with single-agent dactinomycin resulted in an overall 96% remission rate. A total of 20% of patients experienced severe haematological toxicity or mucositis. A further 60% of patients had mild toxicity including hepatotoxicity, serositis, pleurisy or stomatitis.

Single-agent intravenous dactinomycin has also been used with success in patients with and without metastases (Homesley, 1997). One study (Schlaerth *et al*, 1984) showed a cure rate of 82.5% with single-dose biweekly dactinomycin. Patients were changed to 5-day actinomycin or 5-day methotrexate for resistance. Most patients had only mild side effects. Three patients had a rash, two had mild liver function changes and three had thrombocytopenia. Vomiting occurred in 57%. Treatment courses were shorter and less costly in hospital in-patient time. In another study, in nonmetastatic disease, single-dose intravenous dactinomycin 1.25 mg m^−2^ gave a CR rate of 94%. Patients failing to respond adequately were changed to 5-day dactinomycin (Petrilli *et al*, 1987). However, these studies are small, with only 17 and 31 patients, respectively, being evaluated. Larger and longer-term comparative studies will need to be carried out to show superiority over methotrexate, particularly since dactinomycin causes more myelosuppression, nausea and vomiting and alopecia.

In the UK treatment is selected on the basis of risk score. When treatment response was assessed in our low-risk patients, according to whether they were metastatic or nonmetastatic, the CR rate was 60% in the former and 81% in the latter group. The CR rate for metastatic disease is lower than that reported in other smaller series. However, there was no difference in final outcome between these groups since the vast majority of methotrexate-resistant patients responded to second-line chemotherapy (Dobson *et al*, 2000). For all patients, the initial CR rate of 72% and overall 99% cure rate justifies the strategy of treating all low-/medium-risk patients initially with single-agent methotrexate in the knowledge that almost all who are resistant to this first-line therapy can be salvaged with alternative or more intensive regimens. We acknowledge that the etoposide component of our second-line chemotherapy regimen potentially puts these patients at risk of myelodysplastic disorders. Fortunately, we have not observed this complication (Dobson *et al*, 2000); however, we have recently changed our policy, in that methotrexate-failures with low hCG levels now receive dactinomycin alone.

In conclusion, methotrexate achieves high CR rates for low-risk persistent gestational trophoblastic disease. There are several different protocols for treatment administration, but larger comparative studies of their efficacy and toxicity, as well as long-term outcome are needed. However, the UK experience with low-dose intramuscular methotrexate is extensive (Dorreen *et al*, 1988; Bagshawe *et al*, 1989; McNeish *et al*, 2002) and this is a relatively inexpensive and cost beneficial treatment (Hancock, 1997). Our study confirms the efficacy and relative lack of severe short- and long-term toxicity of this regimen. Considering the Charing Cross and Sheffield joint experience for all low-risk patients treated in the UK (735 over 14 years), the cure rate approaches 100%. There does not appear to be an increased risk of second cancers, and fertility is preserved.
